# Current Hospital Topics

**Published:** 1907-03-30

**Authors:** 


### HOSPITAL ADMINISTRATION.

### CONSTRUCTION AND ECONOMICS.

### CURRENT HOSPITAL TOPICS.

**Royal Portsmouth Hospital.**

We are glad to note that the accounts of this hospital are kept upon the uniform system. There has been an increase in the number of in-patients during 1906, the total average number of beds occupied was seven more than in the previous year, whilst the average cost per bed occupied shows a saving of nearly £7 per bed in 1906, as compared with 1905. The annual subscriptions are the largest for four years, as is also the ordinary income, whilst the ordinary expenditure is considerably lower than that of any of the three previous years. One of the townspeople, Mr. George Pratt, has persistently worked for the hospital by raising donations of one penny, and has succeeded in handing over to the hospital funds £140 which he has collected from 18,000 contributors. This instance shows that if individuals are prepared to give a measure of personal service in the cause of the hospitals, no voluntary hospital, which is efficiently managed, need ever be in want of an adequate revenue.

**Addenbrooke's Hospital.**

This hospital has made good progress during the year 1906 owing to the excellent leadership of Mr. A. J. Pell, the Chairman, combined with the zeal and energy which have been brought to bear upon the work by the Secretary-Superintendent, Mr. Richard J. Coles. These good results are attested by the figures published in the report, which show that the cost per head (£5 1s. 0¼d.), is the lowest for six years, whilst the main sources of income, namely annual subscriptions and donations, show a marked increase, the former having grown from £2,372 to £2,852, the total ordinary income, £7,961, in 1906, being the largest for five years. Not only have the annual subscriptions increased, but a new source of income, yielding £412, has been made fruitful by the County Association. We consider these results confer the highest credit upon the management. They prove, as we have always contended, that all classes of the community are prepared to give to this old and famous hospital, when its needs are properly placed before them. Although the average daily number of in-patients was 106 in 1906, compared with 105 in the previous year, the total ordinary expenditure was £80 less. A notable fact in regard to the expenditure is that, by careful supervision, there has been a continuous decrease since 1901 in the expenditure on provisions, surgery and dispensary, and domestic expenses. The economies thus effected have caused a saving of £2,000 as compared with five years ago. We congratulate the Governors upon the marked progress in many directions to which the report bears evidence.

**An Example to be Followed.**

The North Lonsdale Hospital, Barrow-in-Furness, has been the scene of a most interesting work voluntarily undertaken by the men employed at the shipyard of Messrs. Vickers, Sons and Maxim. Experience had shown that if a roomy balcony could be erected for the use of the patients it would minister materially to their welfare and promote their speedy recovery. When this became known the men belonging to all trades in the shipyard asked permission to erect a balcony free of cost. This generous offer was gladly accepted, and with the consent of Messrs. Vickers, Sons and Maxim the different portions of the balcony were first prepared at their yard, where the directors allowed the men to use the machinery to do the work required. When all the parts had been made and put together they were brought to the grounds of the hospital ready for erection. Under the guidance of Mr. Harris, the Architect, the slingers having done their part, the boiler-makers at once fixed the main girders, and they in turn were followed by joiners, bricklayers, plumbers, painters, and labourers, each undertaking his own share of the work. It was customary for the men to assemble at the hospital after work at 5.15 P.M., where tea was provided for them, and where they continued to labour until 8 P.M. The work was commenced in the autumn, and as the evenings drew in the men fixed up electric cables from the *x*-ray department, and continued the work by electric light. So the good work has continued to its completion, which was much expedited by the prolonged fine weather, an advantage the men consider to have been ordered by a Higher Power to help them in their good work. This memorable instance of the gratitude of the artisan class to a hospital for the benefits they have received from it, will no doubt be a pleasure to the patients and to those who took part in the work, when they remember that this balcony has been a voluntary gift to the hospital by some thirty workmen out of the loyalty of their hearts to an institution which has proved so often a good friend to themselves and to members of their families.

